# DWGCN: distance-weighted graph convolutional network for robust spatial domain identification in spatial transcriptomics

**DOI:** 10.3389/fgene.2026.1779455

**Published:** 2026-02-10

**Authors:** Chunfang Peng, Guobin Li, Jiamiao Wu, Qiao Fan, Xiaobo Guo

**Affiliations:** 1 Department of Statistical Science, School of Mathematics, Sun Yat-sen University, Guangzhou, China; 2 Southern China Center for Statistical Science, School of Mathematics, Sun Yat-sen University, Guangzhou, China; 3 Center for Biomedical Data Science, Duke-NUS, Singapore, Singapore

**Keywords:** clustering, graph convolutional networks, representation learning, spatial domain identification, spatial transcriptomics

## Abstract

**Background:**

Graph Convolutional Networks (GCNs) are widely applied for spatial domain identification in spatial transcriptomics (ST), where node representations are learned by aggregating information from neighboring spots. However, most ST workflows construct spatial graphs by assigning equal weights to neighbors and self-loops, and then applying degree-based normalization. This procedure often yields near-uniform adjacency matrices, suppressing natural distance heterogeneity, diminishing spatial resolution, aggravating GCN over-smoothing, and obscuring fine-grained tissue boundaries.

**Methods:**

We introduce DWGCN, a Distance-Weighted Graph Convolutional Network that replaces uniform neighbor assignment with inverse-distance weighting (IDW) and spot-wise normalization. DWGCN enhances locality-sensitive aggregation by assigning larger weights to proximal neighbors, while preserving self-loop dominance to maintain intrinsic spot information and reduce hub-driven dilution.

**Results:**

Across four real and four simulated ST datasets, integrating DWGCN with representative GCN-based frameworks (SEDR, GraphST, SpaNCMG, SpaGIC) generally improved clustering accuracy, particularly in tissues with complex spatial architectures.

**Conclusion:**

These results demonstrate that DWGCN offers a broadly applicable approach for restoring distance-aware structure in spatial graphs, thereby improving the delineation of spatial domain identification.

## 1 Introduction

Spatial transcriptomics (ST) has revolutionized tissue biology research by enabling transcriptome-wide profiling while preserving the spatial context of gene expression (Zhuang, 2021; Larsson et al., 2021). Using diverse imaging- and sequencing-based platforms, ST provides valuable insights into cellular heterogeneity, local microenvironments, and the spatial organization of biological processes within tissues (Chen et al., 2015; Eng et al., 2019; Ståhl et al., 2016; Rodriques et al., 2019). Accurate spatial domain delineation is foundational for reconstructing tissue organization, inferring developmental processes, and resolving disease-associated microenvironments (Xu et al., 2022).

Early computational approaches, such as Seurat (Satija et al., 2015) and spatialLIBD (Pardo et al., 2022), primarily relied on transcriptomic similarity and often overlooked spatial continuity, leading to fragmented or biologically implausible clusters. Subsequent frameworks, including BayesSpace (Zhao et al., 2021) and Giotto (Dries et al., 2021), incorporated spatial priors to improve cluster coherence. However, these methods rely on predefined spatial priors and therefore lack the capacity to learn complex, nonlinear gene–space relationships. In recent years, graph-based deep learning, particularly graph convolutional networks (GCNs), has emerged as a powerful paradigm that simultaneously integrates transcriptomic similarity and spatial proximity. GCN-based spatial identification methods such as SEDR (Xu et al., 2024) and GraphST (Long et al., 2023) have demonstrated superior accuracy and robustness across diverse SRT platforms, highlighting the potential of GCN-based approaches for spatial domain identification.

GCNs build representations by iteratively aggregating information from neighboring spots; consequently, their effectiveness strongly depends on the quality of the underlying adjacency graph (Kipf, 2016). In most GCN-based spatial transcriptomics workflows, the spatial graph is constructed using a K -nearest neighbor (KNN) strategy with equal edge weights, ignoring distance differences and treating all neighbors as equally influential. Edge weights are further rescaled using degree normalization to construct a normalized adjacency matrix (Si et al., 2024; Liu et al., 2024). As a result, the propagation weights become nearly uniform, oversimplifying the underlying spatial structure, where local spatial heterogeneity is diminished, and the contributions of spatially proximal neighbors may be disproportionately diluted. These effects exacerbate the inherent problem of over-smoothing in GCNs, during which repeated propagation under symmetric normalization gradually homogenizes node features and obscures meaningful spatial boundaries (Yang et al., 2020; Rong et al., 2019; Rusch et al., 2023). To partially mitigate over-smoothing, many spatial GCN pipelines restrict the neighborhood size and commonly adopt shallow GCN architectures (Li et al., 2018). Therefore, preserving natural distance heterogeneity in the adjacency matrix is crucial for enhancing the performance of spatial GCN-based models.

**FIGURE 1:**
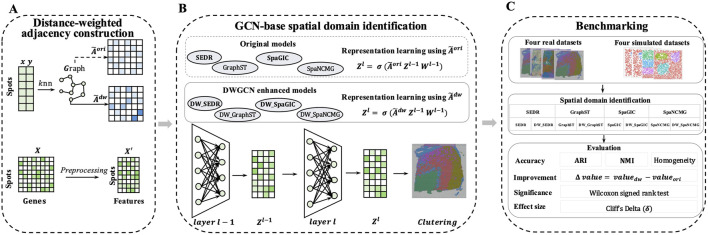
Overview of DWGCN framework. **(A)** Distance-weighted adjacency construction: distance-weighted adjacency matrix ${\tilde{A}}^{dw}$ is constructed using inverse-distance weighting (IDW) over each spot’s top k nearest neighbors, whereas the conventional adjacency matrix ${\tilde{A}}^{\mathrm{ori}}$ is usually constructed with equal-weight edges between nodes. **(B)** GCN-based spatial identification: ${\tilde{A}}^{dw}$ substitutes ${\tilde{A}}^{\mathrm{ori}}$ during GCN-based representation learning in GCN-based spatial clustering frameworks (including SEDR, GraphST, SpaGIC, and SpaNCMG), without altering their original network architectures, yielding DWGCN-enhanced models (including DW_SEDR, DW_GraphST, DW_SpaGIC, and DW_SpaNCMG). **(C)** Benchmarking framework: the performance gains of DWGCN-enhanced models are evaluated on real and simulated datasets, with spatial domain accuracy quantified using ARI, NMI, and Homogeneity.

To address these limitations, we developed DWGCN (a Distance-Weighted Graph Convolutional Network), a framework designed to refine spatial graph construction and improve spatial domain identification (Figure 1). Unlike traditional adjacency matrices with uniform edge weights, DWGCN uses an inverse distance weighting (IDW) scheme to construct adjacency graphs. IDW assigns higher weights to nearby neighbors while keeping each node’s self-loop as the largest, preserving its own information. A tunable exponent parameter p controls the rate of distance decay, enabling adaptation to tissues with diverse local structural patterns. By applying relative rather than absolute distance scaling, DWGCN robustly quantifies spatial similarity across datasets with varying resolutions. Furthermore, instead of degree-based normalization, DWGCN employs spot-wise normalization to preserve the relative ordering of distance-derived weights and prevent hub dominance during message passing. Together, these innovations mitigate over-smoothing, enhance local structural resolution, and improve the biological fidelity of spatial domain delineation.

## 2 Materials and methods

### 2.1 Framework of DWGCN

DWGCN is developed to refine spatial graph construction for spatial transcriptomics (ST) analysis. The workflow of DWGCN consists of two main stages (Figure 1). In the first stage, a sparse, weighted adjacency matrix is constructed using inverse distance weighting (IDW) (Lu and Wong, 2008) over each spot’s neighbors. In the second stage, this distance-weighted adjacency matrix replaces the conventional degree-normalized adjacency matrix in GCN-based spatial clustering frameworks. The performance of DWGCN was systematically evaluated by comparing baseline models with their DWGCN-enhanced versions across both real and simulated spatial transcriptomic datasets.

### 2.2 Distance-weighted graph construction

Let $X\in R^{N\times M}$ denote the gene expression matrix, where N denotes the number of spatial spots and M the number of genes.

#### 2.2.1 Relative distance computation

For any two spots i and j with 2D coordinates $p_{i}$ and $p_{j}$ , the Euclidean distance is computed as shown in Equation 1:

$$d_{ij}^{\left(0\right)}=\|p_{i}-p_{j}{\|}_{2}$$ (1)

To ensure cross-platform consistency across ST technologies with varying resolutions, distances are normalized using the mean nearest-neighbor distance, as defined in Equation 2:

$${\bar{d}}_{\text{min}}=\frac{1}{N}\overset{N}{\underset{i=1}{\sum }}\underset{j\neq i}{\min}d_{ij}^{\left(0\right)},$$ (2)

the relative distance is defined in Equation 3 as:

$$d_{ij}=1+\frac{d_{ij}^{\left(0\right)}}{{\bar{d}}_{\text{min}}}$$ (3)

The additive constant of 1 ensures $d_{ii}=1$ and $d_{ij}>1$ for i≠j , stabilizing inverse-distance computation and preserving the dominance of self-loops during propagation.

#### 2.2.2 Neighborhood graph construction

The relative distances between spots are used to create a neighborhood graph G=(V,E) , where each node i∈V corresponds to a spatial spot, and edges represent spatial proximity. Specifically, top k nearest neighbors for each spot are selected to construct an adjacency matrix, denoted as $A\in R^{N\times N}$ , defined in Equation 4:

$$A_{ij}=\left\{\begin{array}{cc} 1,\; & \text{if }j\in N_{k}\left(i\right),\\ 0,\; & \text{otherwise.} \end{array}\right.$$ (4)

where $N_{k}\left(i\right)$ denotes the set of top k nearest neighbors of node i . Self-loops are explicitly added to A via A←A+I . Note that this adjacency matrix is generally asymmetric, since $j\in N_{k}\left(i\right)$ does not imply $i\in N_{k}\left(j\right)$ . This construction yields an asymmetric KNN graph, which retains directional neighbor relationships rather than enforcing symmetry.

By contrast, many traditional ST workflows symmetrize the graph by setting $A_{ij}=A_{ji}=1$ whenever either node is in the other’s KNN set. Although this simplifies the topology, it may eliminate informative directional structure.

#### 2.2.3 Inverse distance weighting

Edge weights are computed via inverse distance weighting as defined in Equation 5:

$$W_{ij}=\left\{\begin{array}{cc} \frac{1}{d_{ij}^{p}},\; & \text{if }j\in N_{k}\left(i\right)\cup \left\{i\right\},\\ 0,\; & \text{otherwise} \end{array}\right.$$ (5)

here, p≥0 is a tunable exponent controlling the decay rate: larger values of p emphasize spatially close neighbors more strongly. Including the self-node i ensures that intrinsic spot-level information is preserved and generally dominates the message aggregation.

#### 2.2.4 Normalization strategy

To preserve spatial interpretability and avoid undue uniformization, we apply spot-wise row normalization to the distance-weighted adjacency matrix. Given the unnormalized inverse distance weighted adjacency matrix W , we compute the row-normalized distance-weighted adjacency matrix as shown in Equation 6:

$${\tilde{A}}_{ij}^{dw}=\frac{W_{ij}}{\sum_{j\in N_{k}\left(i\right)\cup \left\{i\right\}}W_{ij}}$$ (6)

This operation normalizes each node’s outgoing weights to sum to 1, ensuring that the relative ordering of distance-derived weights is preserved. Row-wise normalization is consistent with widely adopted practices in message-passing Graph Neural Networks (GNNs). In classical spectral graph theory, the random-walk Laplacian $L_{rw}=I-D^{-1}A$ (Von Luxburg, 2007) naturally corresponds to row-normalized propagation, providing a well-established theoretical basis for this normalization strategy.

Thus, our normalization strategy remains fully compatible with standard GCN layers and does not require modifications to training dynamics.

### 2.3 GCN propagation

In a graph convolutional network (GCN), the core operation involves aggregating information from a node’s local neighborhood based on a normalized adjacency matrix. Let $Z^{l}\in R^{N\times d^{l}}$ denote the node representations at layer l , the layer-wise propagation rule is formally defined in Equation 7 as:

$$Z^{l}=\sigma \left(\tilde{A}Z^{l-1}W^{l-1}\right)$$ (7)

where $W^{l-1}$ is a learnable weight matrix, σ(⋅) is a nonlinear activation, and $\tilde{A}\in R^{N\times N}$ is the normalized adjacency matrix.

Spatial GCN frameworks typically construct a KNN graph with a binary adjacency matrix A . The degree matrix D is defined as $D_{ii}=\sum_{j}A_{ij}$ . Applying symmetric degree normalization yields the original normalized adjacency matrix as shown in Equation 8:

$${\tilde{A}}^{\mathrm{ori}}=D^{-\frac{1}{2}}AD^{-\frac{1}{2}}$$ (8)

In DWGCN, the original degree-normalized adjacency matrix ${\tilde{A}}^{\mathrm{ori}}$ is replaced with the proposed distance-weighted adjacency matrix ${\tilde{A}}^{dw}$ (as described in Section 2.2), which assigns larger weights to spatially closer spots. The propagation rule is defined in Equation 9 as:

$$Z^{l}=\sigma \left({\tilde{A}}^{dw}Z^{l-1}W^{l-1}\right)$$ (9)

### 2.4 Integration into existing spatial GCN workflows

DWGCN replaces the original spatial adjacency in GCN-based frameworks with the distance-weighted adjacency matrix ${\tilde{A}}^{dw}$ while leaving all model architectures, losses, and hyperparameters unchanged. This design isolates the effect of graph construction, enabling fair and architecture-independent benchmarking of distance-aware connectivity.

Beyond providing a controlled comparison setting, the resulting ${\tilde{A}}^{dw}$ functions as a generalizable graph foundation that can be readily incorporated into diverse spatial transcriptomic workflows. Although broadly applicable to tasks such as spatial domain identification, trajectory inference, visualization, and denoising, here we focus specifically on evaluating its impact on spatial domain identification across representative GCN-based models.

### 2.5 Benchmarking and evaluation

We benchmarked DWGCN against four graph-based deep learning frameworks for spatial domain identification (Supplementary Table S1): SEDR (Xu et al., 2024), GraphST (Long et al., 2023), SpaNCMG (Si et al., 2024), and SpaGIC (Liu et al., 2024). For each baseline method, we followed the official implementations and default parameter settings unless otherwise specified. In the DWGCN-enhanced models, the original degree-normalized adjacency matrix ${\tilde{A}}^{\mathrm{ori}}$ was replaced with the distance-weighted adjacency matrix ${\tilde{A}}^{dw}$ (with k = 12 and p = 2), and all other hyperparameters were kept unchanged to ensure a fair comparison.

Benchmarking was conducted on eight datasets, including four real and four simulated collections. The real-world collection comprised four publicly available spatial transcriptomics datasets with curated domain annotations (Supplementary Table S2): 12 slices of human dorsolateral prefrontal cortex (DLPFC) (Pardo et al., 2022), one mouse brain sagittal anterior section (Mouse_Brain) (Ji et al., 2020), one human breast cancer Block A Section 1 (Human_Breast) (Ji et al., 2020), and three mouse embryos (Mouse_Embryos) of E9.5-stage (Chen et al., 2022). In addition, four *in silico* datasets were generated using simSRT (Zhu et al., 2023) to model tissues containing 3, 5, 8, and 10 spatial domains (referred to as the cluster_3 to cluster_10 datasets; Supplementary Table S3). Each simulated dataset contained eight independently generated samples to account for stochastic variability and ensure statistical robustness. Details of data preprocessing and normalization are provided in the Supplementary Material.

Clustering accuracy was evaluated using three complementary metrics: Adjusted Rand Index (ARI) (Hubert and Arabie, 1985), Normalized Mutual Information (NMI) (Strehl and Ghosh, 2002), and Homogeneity (Rosenberg and Hirschberg, 2007). For each sample, both the baseline and DWGCN-enhanced models were run 20 times to account for randomness in model training. Performance improvement was evaluated using paired run-wise differences between the two models. For each sample s and run r , the improvement was computed as $\Delta value_{s,r}=value_{s,r}^{dw}-value_{s,r}^{\mathrm{ori}}$ , where $value_{s,r}^{dw}$ and $value_{s,r}^{\mathrm{ori}}$ denote the metric values of the DWGCN-enhanced and baseline models, respectively. For summary reporting, run-wise differences were averaged within each sample.

The statistical significance of the paired differences was assessed using the paired Wilcoxon signed-rank test (Bauer, 1972). Multiple-testing correction was performed using the Benjamini–Hochberg procedure (Benjamini and Hochberg, 1995), and the resulting adjusted p-values are reported as FDR values. Unless otherwise stated, differences were considered statistically significant at FDR <0.05 . In addition, effect sizes for the paired comparison were quantified using Cliff’s Delta (δ) , which measures the probability that a randomly selected value from the DWGCN-enhanced method exceeds that from the baseline (Cliff, 2014).

Analyses were conducted at both the sample and dataset levels. Sample-level analyses enabled direct paired comparison within each biological replicate, controlling for intra-dataset variability. Dataset-level analyses aggregated sample-level statistics to evaluate whether DWGCN yielded consistent, statistically significant improvements across heterogeneous biological and technical contexts. Together, this multi-level paired statistical design provided a robust and interpretable assessment of the performance enhancement introduced by DWGCN.

## 3 Performance of DWGCN

### 3.1 DWGCN generates distance-aware neighborhood weights

To examine how DWGCN reshapes the local aggregation structure, we compared the normalized edge-weight profiles generated by DWGCN ( k = 12) with those produced by a degree-normalized KNN adjacency constructed using symmetric normalization. For reference, degree normalization is representative of the normalization schemes widely adopted in standard GCNs, and is therefore used here as a conventional baseline. As summarized in Supplementary Table S4, the conventional adjacency construction produces nearly uniform weights (self-loop ≈0.077 ; neighbor weights ≈0.064 –0.077), indicating that spatial distance exerts minimal influence on the aggregation process. Because conventional KNN adjacency assigns all connected neighbors an identical weight before normalization, degree normalization cannot recover the lost geometric information. This near-uniform weighting reflects the smoothing-dominant behavior of conventional GCN propagation, where symmetric normalization reduces geometric distinctions among neighbors.

DWGCN, in contrast, introduces distance-aware weighting that becomes progressively more localized as the distance exponent p increases. When p=0 , DWGCN produces uniform weights within each node’s KNN neighborhood, although the resulting graph is naturally asymmetric due to directional relative distances. For moderate exponents ( p≈0.5 –1), the self-loop weight increases, and closer neighbors consistently receive higher weights than farther ones, establishing meaningful spatial discrimination while still retaining contributions from all neighbors. For large exponents (p≥4) , the distribution becomes highly concentrated: the self-loop weight dominates, and distant neighbors contribute only marginally (on the order of $10^{-3}$ or lower). The parameter p continuously controls the strength of spatial smoothing, ranging from uniform averaging (p=0) to distance-sensitive, structure-preserving aggregation.

Rather than seeking the optimal hyperparameters, which can vary across datasets and downstream tasks, we adopt a representative and stable configuration ( k=12 , p=2 ) to illustrate DWGCN’s intended behavior. At p=2 , the resulting weight profile (self-loop ≈0.36 ; nearest neighbors ≈0.09 ; distant neighbors decreasing smoothly to ≈0.03 ) reflects a balanced regime in which self-information is strengthened and spatially proximate neighbors are emphasized, while farther neighbors still retain non-negligible influence.

### 3.2 Benchmarking results of real datasets

**FIGURE 2:**
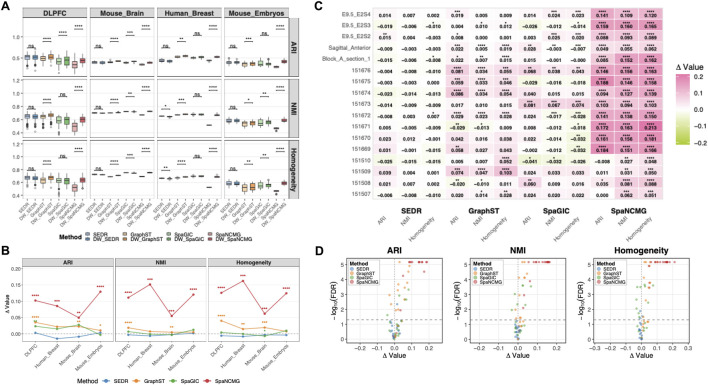
Performance of DWGCN-enhanced methods on real datasets. **(A)** Boxplots summarizing clustering performance across real datasets using three evaluation metrics: Adjusted Rand Index (ARI), Normalized Mutual Information (NMI), and Homogeneity. **(B)** Line plots showing the average improvement in clustering performance induced by DWGCN across datasets. Each subplot corresponds to an evaluation metric, and each line represents a clustering framework. **(C)** Heatmap illustrating sample-level performance gains, where color intensity denotes the magnitude of improvement (difference between DWGCN-enhanced and baseline model scores). Significance levels are denoted as: FDR <0.0001(****),<0.001(***),<0.01(**),<0.05(*),and≥0.05(ns) . **(D)** Volcano plots depicting the relationship between the magnitude of improvement and its statistical significance. The x-axis indicates the mean improvement, and the y-axis shows the log-transformed FDR from the paired Wilcoxon signed-rank test.

Across four real spatial transcriptomics datasets, incorporating DWGCN consistently improved clustering accuracy under diverse model architectures and biological contexts. As summarized in Figure 2A, the DWGCN-enhanced versions outperformed their baseline counterparts on all three evaluation metrics (Adjusted Rand Index (ARI), Normalized Mutual Information (NMI), and Homogeneity), yielding average performance gains of 0.038 (9.09%), 0.032 (5.44%), and 0.038 (6.30%), respectively. Visual inspection of spatial clustering patterns further corroborated these trends: DWGCN-enhanced models produced clearer laminar structures on representative real datasets (Supplementary Figure S1).

At the dataset level, the accuracy improvement provided by DWGCN was consistent across all four datasets, demonstrating stable performance gains regardless of tissue type or experimental condition (Figure 2B). Across four datasets and three clustering metrics, a total of 48 paired comparisons were performed between each baseline model and its DWGCN-enhanced version. Among them, 27 cases (56.3%) exhibited significant enhancement, whereas only three (6.3%) showed significant decreases (Supplementary Table S5). Effect-size analysis showed consistent trends, with positive large effect sizes in 23 (47.9%) cases and negative large effect sizes in four (8.3%) cases (Supplementary Table S6).

On a per-sample basis, 75%, 67.65%, and 69.12% of samples exhibited improved performance, approximately 50% reached statistical significance (Supplementary Table S7). In comparison, only 4%–10% of samples exhibited significant declines. The heatmap in Figure 2C further visualizes sample-level performance gains, where color intensity reflects the magnitude of improvement. Sample-wise distributions of ARI, NMI, and Homogeneity also consistently favored DWGCN across methods (Supplementary Figure S2), with performance gains consistently observed across individual samples (Supplementary Figure S3).

To further characterize model-specific behavior, volcano plots highlighted distinct sensitivity patterns among the four representative models (Figure 2D). Overall, DWGCN yielded the greatest enhancement when integrated with SpaNCMG, followed by GraphST, SpaGIC, and SEDR. DWGCN achieved 12 and 11 significant improvements for SpaNCMG and GraphST, respectively, without any observed degradation. SpaNCMG displayed the most stable behavior, showing 100% significant gains based on 12 dataset–metric combinations. GraphST exhibited slightly lower but still consistent improvement with 80%–85% significant improvements and no negative cases. In contrast, SpaGIC exhibited both positive and negative effects, with roughly half of its comparisons showing significant improvements and the remainder showing significant decreases. SEDR showed minimal response to DWGCN, with only marginal decreases in the Human_Breast dataset for NMI and Homogeneity, suggesting that its embedding structure is less influenced by distance-weighted connectivity.

### 3.3 Benchmarking based on simulated datasets

**FIGURE 3:**
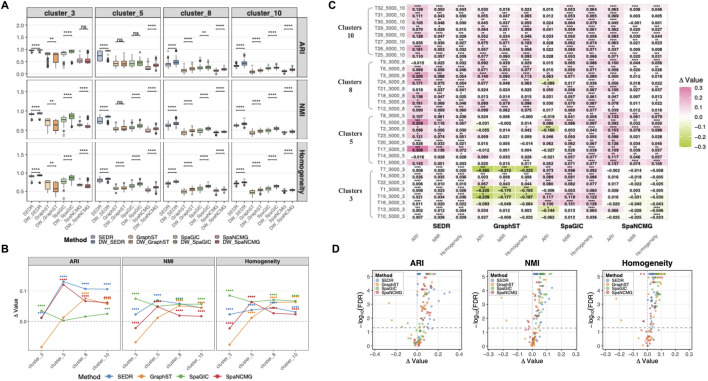
Evaluation of DWGCN-enhanced methods on simulated datasets. **(A)** Boxplots summarizing clustering accuracy across simulated datasets, evaluated using ARI, NMI, and Homogeneity. Comparison of clustering performance for representative simulated samples across original and DWGCN-enhanced methods. **(B)** Line plots displaying the mean improvement trends of DWGCN across all simulated scenarios. **(C)** Heatmap illustrating dataset-level performance improvements, where the color gradient reflects the relative effect size of DWGCN over baseline methods. Significance levels are denoted as: FDR <0.0001(****),<0.001(***),<0.01(**),<0.05(*),and≥0.05(ns) . **(D)** Volcano plots showing the relationship between the effect magnitude and statistical significance of improvements in simulated settings. Each point represents one simulated dataset, with the x-axis indicating the average DWGCN-induced gain and the y-axis representing the log-transformed FDR.

Performance evaluation on simulated datasets revealed an even more pronounced improvement achieved by DWGCN across all tested frameworks (Figure 3). As shown in Figure 3A, the DWGCN-enhanced versions consistently outperformed their baseline counterparts across three metrics. The average gains over the baseline methods were 0.047 (an 11.46% improvement relative to the baseline mean ARI) for ARI, 0.033 (5.66%) for NMI, and 0.036 (5.67%) for Homogeneity. A large majority of samples (84.38%, 89.06%, and 88.28% for ARI, NMI, and Homogeneity) showed improved performance, of which approximately 57.81%–72.66% reached statistical significance (Supplementary Table S10, Supplementary Figure S5). The proportion of samples with decreased performance remained low (10%–15%), and significant decreases were minimal (3%–6%). Representative examples from simulated datasets also illustrated the improved recovery of ground-truth spatial structure under DWGCN (Supplementary Figure S4).

At the dataset level, DWGCN outperformed baseline models in 89.6% (43/48) of pairwise comparisons (Figure 3B). Statistical significance was achieved in 81.3% (39/48) of cases, and 70.8% (24/48) showed large effect sizes (Supplementary Tables S8, S9). In contrast, only 10.4% (5/48) exhibited significant decreases, and 8.3% (4/48) showed no difference. Consistently, the sample-level heatmap in Figure 3C illustrated that 87.2% (335/384) of individual samples showed higher accuracy under DWGCN, with 66.1% achieving significant improvements. The volcano plot in Figure 3D further confirmed this pattern, with most points shifted toward the positive-effect side, indicating predominantly beneficial and frequently significant improvements. Together, these results demonstrate that the observed enhancement is both statistically significant and a large-effect improvement across dataset- and sample-level analyses.

Notably, the performance benefit of DWGCN became more pronounced on datasets with higher clustering complexity. As shown in Figure 3A, clustering performance declined as the number of clusters increased, reflecting the increasing difficulty of resolving finer-grained spatial boundaries as cluster numbers grow. However, DWGCN-enhanced models maintained higher clustering accuracy and demonstrated progressively stronger benefits as the number of spatial domains increased from 3 to 10 (Supplementary Figure S6). At low complexity (3 clusters), improvements were modest and occasionally unstable, with slight declines observed for GraphST and SpaNCMG. In contrast, at higher complexity (5–10 clusters), all frameworks achieved consistent and substantial performance gains. Effect size analysis confirmed this trend: the average Cliff’s Delta increased steadily from 0.272 (small effect) at cluster_3 setting, to 0.575 (large effect) at cluster_5, 0.760 (large effect) at cluster_8, and 0.816 (large effect) at cluster_10 (Supplementary Table S10).

Collectively, these findings indicate that DWGCN not only improves clustering accuracy but also enhances model robustness and scalability under increasingly complex spatial structures, underscoring its strong potential to generalize across diverse spatial transcriptomics settings.

## 4 Discussion

In this study, we present DWGCN, a distance-weighted graph convolutional framework that enhances spatial domain identification by refining the construction of spatial adjacency. GCN-based spatial models aggregate information from graph neighbors, making their performance tightly dependent on the fidelity of the adjacency matrix. However, conventional binary KNN adjacency followed by degree normalization in many ST pipelines assigns almost uniform neighbor weights, suppressing natural distance heterogeneity and diminishing distance-dependent variation during message passing. This tendency further amplifies the intrinsic over-smoothing behavior of GCNs, potentially obscuring fine or irregular domain boundaries.

DWGCN reintroduces distance heterogeneity into graph construction, thereby breaking the uniform propagation pattern and mitigating excessive feature averaging. The dominance of the self-loop ensures that spot-specific information remains the primary signal throughout propagation. DWGCN achieves distance-aware adjacency refinement through three coordinated mechanisms. First, inverse-distance weighting (IDW) with a tunable decay exponent p emphasizes proximal neighbors and restores distance decay patterns commonly observed in spatial transcriptomics. Second, relative distance scaling within each tissue section ensures comparability of distance-derived weights across platforms with distinct spatial resolutions. Third, replacing global degree normalization with local row-wise normalization preserves within-spot weight ratios and prevents hub dominance. Together, these mechanisms produce adjacency matrices that retain biologically plausible spatial heterogeneity, reflecting the expectation that nearby spots tend to share transcriptomic similarity and belong to the same cell type.

Our benchmarking results across four real and four simulated datasets demonstrate that DWGCN yields performance gains in the majority of sample-level comparisons. Improvements were most pronounced in simulated datasets with complex spatial architectures or fine-grained transitions, where distance-aware weighting more effectively captures local structural continuity. These results suggest that DWGCN is particularly beneficial in scenarios where domain boundaries are subtle. In contrast, datasets with coarse and well-separated domains showed smaller improvements, likely because their broad homogeneous regions already limit the adverse effects of uniform neighbor weighting. These observations highlight the importance of selecting an appropriate decay exponent p in accordance with the spatial granularity of the tissue.

Performance differences across frameworks further reveal that adjacency refinement interacts with model architecture. SpaNCMG exhibited the largest and most consistent improvements after integration with DWGCN, likely because its multi-view graph reconstruction and attention-based fusion make it more sensitive to the quality of spatial graphs. In comparison, SEDR, GraphST, and SpaGIC showed more dataset-dependent gains, reflecting the fact that adjacency refinement constitutes only one component within their broader architectures. We also observed larger improvements in simulated datasets, which typically have uniform spot densities and lower noise, conditions under which distance heterogeneity is more clearly reflected in the weighted adjacency. Real tissues inherently contain morphological irregularities and measurement noise, leading to more moderate but still predominantly positive gains. Taken together, these results indicate that DWGCN offers broadly beneficial enhancements, with the magnitude of improvement shaped by both model architecture and dataset characteristics.

DWGCN has several limitations and practical considerations. The choice of p and k controls the trade-off between locality and connectivity. While default settings ( k=12 , p=2 ) provided stable performance in our benchmarks, optimal configurations depend on spot density, tissue scale, and the downstream model. Additional validation across more platforms and tissue types will be needed to establish robust parameter recommendations. Future work may focus on adaptive strategies for selecting k and p , incorporating learned distance functions, and extending distance-aware graph construction to multimodal or time-resolved spatial omics.

In summary, DWGCN provides a biologically motivated and implementation-friendly strategy for restoring distance sensitivity in spatial GCN pipelines. By embedding distance awareness directly into graph construction, it mitigates excessive homogenization and improves domain delineation, particularly in tissues requiring high spatial resolution.

## Data Availability

The original contributions presented in the study are included in the article/Supplementary Material, further inquiries can be directed to the corresponding author.
