# Supplementary material for: DWGCN: distance-weighted graph convolutional network for robust spatial domain identification in spatial transcriptomics
Source: Front Genet. 2026 Feb 10;17:1779455. doi: 10.3389/fgene.2026.1779455 (PMC12928605; doi:10.3389/fgene.2026.1779455)
Supplement: Supplementary file 1 [file DataSheet1.docx]

**DWGCN: Distance-Weighted Graph Convolutional Network for Robust Spatial Domain Identification in Spatial Transcriptomics**

# Materials and Methods

## Spatial domain identification methods

We included four GCN-based deep learning methods for spatial domain identification in our benchmarking study: **SEDR**, **GraphST**, **SpaGIC**, and **SpaNCMG**.

### SEDR

SEDR learns a low-dimensional latent representation of gene expression embedded with spatial information for spatial transcriptomics analysis. The top 2,000 highly variable genes were selected using the Scanpy package, and dimensionality reduction was performed using PCA (200 components). The spatial neighborhood graph was constructed with k = 6 neighbor. The model was trained for 200 epochs with a learning rate of 0.01, and the parameter using_dec was set to False.
The DWGCN-enhanced version, **DW_SEDR**, replaces the original degree-normalized adjacency matrix with the distance-weighted (DW) graph to better capture relative spatial distances and neighbor strength. For DW_SEDR, k = 12 and p = 2, while all other parameters remained identical to the default configuration.

### GraphST

GraphST combines graph neural networks with self-supervised contrastive learning to learn discriminative spot representations by minimizing embedding distance between spatially adjacent spots and maximizing it between distant ones. The top 2,000 highly variable genes were selected using Scanpy.pp.highly_variable_genes, and PCA (200 components) was applied for dimensionality reduction. As in the original paper, the spatial graph was constructed using k = 3 nearest neighbors. The model was trained for 200 epochs, and post-training embeddings (emb_pca) were clustered using the mclust package in R.
The **DW_GraphST** variant incorporated the distance-weighted (DW) adjacency matrix instead of the default degree-normalized graph, with k = 12 and p = 2. All other hyperparameters were consistent with the original settings.

### SpaGIC

SpaGIC (Spatial Graph Information Clustering) integrates gene expression and spatial proximity through dual-channel GCN encoders and self-supervised contrastive learning. The top 3,000 highly variable genes were selected using Scanpy, and a spatial KNN graph was constructed with k = 5 (default). The encoder consists of two GCN layers with 512 and 64 neurons, respectively, trained for 200 epochs using the Adam optimizer (learning rate = 0.001, weight decay = 0.01). The loss function includes three weighted components (λ₁ = 60, λ₂ = 0.01, λ₃ = 0.01).
The DWGCN-enhanced version, **DW_SpaGIC**, followed the same architecture and optimization scheme but substituted the degree-normalized adjacency matrix with the distance-weighted (DW) graph (k = 12, p = 2), to improve spatial sensitivity and achieve smoother neighborhood transitions

### SpaNCMG

SpaNCMG (Spatial Neighborhood-Complementary Mixed-Graph) achieves precise spatial domain identification through a mixed-view GCN framework that integrates local KNN and global *r*-radius structures. The model fuses expression, spatial, and morphological information using an attention-based adaptive module and employs kernel PCA for dimensionality reduction. The official implementation (v1.0) was used with three graph channels (spatial, expression, morphology), embedding dimension = 64, temperature coefficient $\tau$ = 0.1, and the Adam optimizer (learning rate = 1e–3). The model was trained for 200 epochs, with all other parameters set to default.
In the **DW_SpaNCMG** variant, the degree-normalized adjacency was replaced by the distance-weighted (DW) graph (*k = 12*, *p = 2*), while keeping all other configurations unchanged.

## Benchmarking datasets

### Real datasets

We evaluated DWGCN on four publicly available spatial transcriptomics (ST) datasets with manually curated spatial domain annotations. The human dorsolateral prefrontal cortex (DLPFC) dataset contains 12 tissue slices, each comprising 3,460–4,789 spots with an average of 12,474 detected genes, with annotations provided by the original authors. The mouse brain sagittal anterior section (Mouse_Brain) dataset consists of 2,695 spots and 14,557 detected genes, while the human breast cancer Block A Section 1 (Human_Breast) dataset contains 3,798 spots and 16,280 detected genes. Both the Mouse_Brain and Human_Breast datasets using the 10x Visium platform, and their manual annotations were obtained from the STimage-1K4M resource. The mouse embryos (Mouse_Embryos) dataset was generated with the Stereo-seq platform and consists of three slices from E9.5-stage mouse embryos.

### Simulated datasets

We further generated four simulated datasets using simSRT. Simulation parameters varied the number of cell types (3, 5, 8, 10), number of cells (3,000 or 5,000), expression dispersion (0.5 or 1.0), and mean expression level (1 or 2), while gene composition (including 1,000 highly expressed, 2,000 moderately expressed, 2,000 noise genes) and dropout rate (0.05) were fixed. Since the number of cell-types has the strongest impact on spatial domain identification, simulated datasets were grouped by the number of ground-truth categories into four scenario groups: cluster_3, cluster_5, cluster_8, and cluster_10.

For all datasets, genes detected in fewer than 50 spots or with fewer than 10 counts were removed. Preprocessing followed the recommendations in each method’s original publication to ensure fair comparison. This setup allowed systematic evaluation of how spatial domain identification complexity affects model performance.

## Evaluation metrics of clustering accuracy

To quantitatively evaluate the clustering performance of different methods, three clustering metrics were employed: **Adjusted Rand Index (ARI)**, **Normalized Mutual Information (NMI)**, and **Homogeneity**. These metrics measure the agreement between the predicted clustering results and the ground-truth labels from different perspectives.

### Adjusted Rand Index (ARI)

The Adjusted Rand Index (ARI) measures the similarity between two clustering, corrected for chance. The ARI is defined as:

$$ARI= \frac{(a - (a + b) * (a + c)/(a + b + c + d))}{((a + b + a + c)/2 - (a + b) * (a + c)/(a + b + c + d))}$$

Where a represents the number of pairs clustered together in both x and y, b represents the number of pairs clustered together in x but not in y, c represents the number of pairs clustered together in y but not in x, and d represents the number of pairs clustered apart in both x and y. The Adjusted Rand Index (ARI) ranges from -1 to 1, where 1 indicates perfect agreement, 0 indicates random clustering, and negative values suggest worse-than-random clustering.

### Normalized Mutual Information (NMI)

The Normalized Mutual Information measures the mutual dependence between the predicted clusters $C$and the ground-truth labels $L$. It is based on information theory and normalized to ensure comparability across datasets.

$$\text{NMI}(C,L)=\frac{2\times I(C;L)}{H(C)+H(L)}$$

where

$$I\left( C;L \right)=\sum_{i=1}^{\mid C\mid} \sum_{j=1}^{\mid L\mid} P(i,j)log\frac{P(i,j)}{P(i)P(j)}$$

is the mutual information between the two partitions, and

$$H\left( C \right)=-\sum_{i=1}^{\mid C\mid} P\left( i \right)\log P\left( i \right), H(L)=-\sum_{j=1}^{\mid L\mid} P(j)logP(j)$$

are the entropies of $C$and $L$, respectively. NMI ranges from 0 (no mutual information) to 1 (perfect correspondence). It captures how much information about the true clusters is preserved in the predicted partition and is particularly useful when the number of clusters differs between the two label sets.

### Homogeneity

Homogeneity assesses whether each cluster contains only members of a single class. It is defined based on conditional entropy:

$$h=1-\frac{H(L\mid C)}{H(L)}$$

where

$$H\left( L \mid C \right)=-\sum_{i=1}^{\mid C\mid} \sum_{j=1}^{\mid L\mid} P(i,j)log\frac{P(i,j)}{P(i)}$$

is the conditional entropy of the true labels given the predicted clusters.

Homogeneity ranges from 0 (completely mixed clusters) to 1 (perfectly pure clusters). Unlike ARI and NMI, Homogeneity focuses solely on intra-cluster label purity and does not penalize over-segmentation, thus complementing ARI and NMI in detecting different types of clustering bias.

Together, ARI, NMI, and Homogeneity provide a comprehensive evaluation of clustering performance from orthogonal perspectives: ARI emphasizes pairwise label agreement and penalizes both over- and under-clustering; NMI measures information preservation between partitions; and Homogeneity quantifies label purity within clusters.

By jointly analyzing these metrics, we ensure that DWGCN enhancement is consistently validated in terms of clustering accuracy, information fidelity, and internal consistency across datasets.

## Paired statistical design and evaluation procedure

To rigorously evaluate the performance improvement brought by DWGCN, we designed a comprehensive **paired comparative analysis** framework between each baseline clustering algorithm and its DWGCN-enhanced counterpart: SEDR vs. DW_SEDR, GraphST vs. DW_GraphST, SpaGIC vs. DW_SpaGIC, and SpaNCMG vs. DW_SpaNCMG.
This paired design ensures that each comparison is made on the same biological sample under identical conditions, thereby controlling for inter-sample variability and attributing any performance differences solely to the DW enhancement.

For each sample, both the baseline and DWGCN-enhanced models were run 20 times to account for randomness in model training. Three clustering evaluation metrics were computed by comparing the predicted domain labels with the annotated ground truth, including **Adjusted Rand Index (ARI), Normalized Mutual Information (NMI),** and **Homogeneity**. Performance improvement was evaluated using paired run-wise differences between the two models. For each sample $s$ and run $r$, the **paired performance difference** between the DWGCN-enhanced and baseline methods is:

$${\Delta value}_{s,r}= {\Delta value}_{s,r}^{dw}- {\Delta value}_{s,r}^{ori}$$

where ${\Delta value}_{s,r}^{dw}$ and ${\Delta value}_{s,r}^{ori}$ denote the metric value (e.g., ARI) of the DWGCN-enhanced and baseline methods, respectively.

Because performance metrics may not be normally distributed, statistical significance was assessed using the **Wilcoxon signed-rank test** (implemented via wilcox.test in R), a non-parametric paired test suitable for our repeated-measure design. To correct for inflated false discovery rates arising from multiple comparisons across datasets, methods, and metrics, **Benjamini–Hochberg (BH)** correction was applied to all p-values, controlling the False Discovery Rate. *FDR* (adjusted p-value) was interpreted as follows: *FDR* < 0.0001 as "****", *FDR* < 0.001 as "***", *FDR* < 0.01 as "**", *FDR* < 0.05 as "*", and *FDR* ≥ 0.05 as "ns". This pair-method paired analysis design ensured statistical rigor by controlling for inter-sample variation and enabling direct comparison of performance across methods for the same sample, thereby accurately assessing the improvement effects of DW-GCN.

To further characterize the **magnitude** of performance improvement, we calculated **Cliff’s Delta (**$\boldsymbol{\delta}$**)**, a robust non-parametric effect size measure. As a non-parametric effect size metric, Cliff’s Delta quantifies the probability that a randomly selected ARI value from the DWGCN-enhanced method exceeds that from the baseline:

$$\boldsymbol{\delta=}\frac{\boldsymbol{\#}\left( \boldsymbol{X>Y} \right)\boldsymbol{-\#(X<Y)}}{\boldsymbol{n}_{\boldsymbol{X}}\boldsymbol{n}_{\boldsymbol{Y}}}$$

where $\boldsymbol{X}$ and $Y$ denote paired values from the DWGCN enhanced and baseline methods, respectively, and $\boldsymbol{n}_{\boldsymbol{X}}$​ and $\boldsymbol{n}_{\boldsymbol{Y}}$ are their sample sizes. $\boldsymbol{\delta}$ ranges from -1 to 1, with values closer to 1 or -1 indicating stronger positive or negative effects, respectively. According to standard interpretation, |δ| < 0.15、0.15 ≤ |δ| < 0.33、0.33 ≤ |δ| < 0.47 and |δ| ≥ 0.47 correspond to negligible, small, medium, and large effect sizes, respectively.

All analyses were performed at **two complementary levels**:

1. **Sample-level analysis:** paired tests were conducted within each sample to assess whether DWGCN consistently improved clustering for individual biological replicates.
2. **Dataset-level analysis:** paired metric values were aggregated across all samples of the same dataset, providing an overall evaluation of whether DWGCN yielded statistically consistent improvements across the dataset.

This multi-level paired analysis, combined with non-parametric statistical testing and effect size estimation, ensured a rigorous and interpretable assessment of the performance gains introduced by the DWGCN framework.

# Supplementary Tables

**Supplementary Table 1：GCN-based spatial domain identification approaches**

| Method | GCN-based | Representation Learning Framework | Loss Function | k (Neighbors) | Adjacency Matrix Type | Normalization |
| --- | --- | --- | --- | --- | --- | --- |
| SEDR | Yes | GCN encoder + Auto-encorder | Reconstruction loss | 6 | Symmetric, unweighted | Degree Matrix |
| GraphST | Yes | GCN encoder + Corrupted graph | Reconstruction loss +  Contrastive loss | 3 | Symmetric, unweighted | Degree Matrix |
| SpaGIC | Yes | GCN encoder | Reconstruction loss | 5 | Symmetric, unweighted | Degree Matrix |
| SpaNCMG | Yes | GCN encoder + Multi-view + self-attention fusion | Reconstruction loss +  Contrastive loss | 12 | Symmetric, unweighted | Degree Matrix |

**Supplementary Table 2：Real datasets**

| Dataset | Sample.No | #Cluster | #Unit | #Gene | Sparsity | Gene.exp |
| --- | --- | --- | --- | --- | --- | --- |
| DLPFC | 151507 | 8 | 4226 | 11,982 | 0.885 | 0.214 |
| DLPFC | 151508 | 8 | 4384 | 11,452 | 0.898 | 0.183 |
| DLPFC | 151509 | 8 | 4789 | 12,407 | 0.886 | 0.204 |
| DLPFC | 151510 | 8 | 4634 | 12,094 | 0.889 | 0.201 |
| DLPFC | 151669 | 6 | 3661 | 12,330 | 0.856 | 0.304 |
| DLPFC | 151670 | 6 | 3498 | 11,948 | 0.863 | 0.285 |
| DLPFC | 151671 | 6 | 4110 | 12,811 | 0.857 | 0.303 |
| DLPFC | 151672 | 6 | 4015 | 12,491 | 0.861 | 0.289 |
| DLPFC | 151673 | 8 | 3639 | 13,104 | 0.834 | 0.348 |
| DLPFC | 151674 | 8 | 3673 | 14,001 | 0.809 | 0.420 |
| DLPFC | 151675 | 8 | 3592 | 12,462 | 0.857 | 0.286 |
| DLPFC | 151676 | 8 | 3460 | 12,604 | 0.849 | 0.305 |
| Mouse_Brain | Sagittal_Anterior | 52 | 2695 | 14,557 | 0.598 | 1.764 |
| Human_Breast | Block_A_section_1 | 20 | 3798 | 16,280 | 0.656 | 1.338 |
| Mouse_Embryos | E9.5_E2S2 | 13 | 4356 | 14,117 | 0.781 | 0.240 |
| Mouse_Embryos | E9.5_E2S3 | 13 | 5059 | 14,847 | 0.782 | 0.240 |
| Mouse_Embryos | E9.5_E2S4 | 13 | 5,797 | 13,787 | 0.832 | 0.174 |

Note: Sample No. : Sample number; #Cluster: Average number of clusters per sample; #Unit: Average sequenced units (e.g., cells or spots) per sample; #Gene: Average number of sequenced genes per sample; Gene.exp: Average mRNA counts per sequenced unit and gene.

**Supplementary Table 3：Simulated datasets**

| Dataset | Sample.No | #Cluster | #Unit | #Gene | Sparsity | Gene.exp |
| --- | --- | --- | --- | --- | --- | --- |
| cluster_3 | T1_3000_3 | 3 | 2965 | 5,000 | 0.306 | 1.927 |
| cluster_3 | T13_3000_3 | 3 | 3065 | 5,000 | 0.486 | 0.950 |
| cluster_3 | T7_3000_3 | 3 | 2965 | 5,000 | 0.385 | 1.946 |
| cluster_3 | T19_3000_3 | 3 | 2965 | 5,000 | 0.542 | 0.971 |
| cluster_3 | T4_5000_3 | 3 | 4955 | 5,000 | 0.316 | 1.958 |
| cluster_3 | T16_5000_3 | 3 | 4955 | 6,000 | 0.486 | 0.966 |
| cluster_3 | T10_5000_3 | 3 | 4955 | 5,000 | 0.380 | 1.932 |
| cluster_3 | T22_5000_3 | 3 | 4955 | 5,000 | 0.542 | 0.981 |
| cluster_5 | T2_3000_5 | 5 | 2965 | 5,000 | 0.323 | 2.096 |
| cluster_5 | T14_3000_5 | 5 | 2965 | 5,000 | 0.497 | 1.038 |
| cluster_5 | T8_3000_5 | 5 | 2965 | 5,000 | 0.393 | 2.057 |
| cluster_5 | T20_3000_5 | 5 | 2965 | 5,000 | 0.547 | 1.016 |
| cluster_5 | T5_5000_5 | 5 | 4955 | 5,000 | 0.325 | 2.108 |
| cluster_5 | T17_5000_5 | 5 | 4955 | 5,000 | 0.496 | 1.038 |
| cluster_5 | T11_5000_5 | 5 | 4955 | 5,000 | 0.396 | 2.078 |
| cluster_5 | T23_5000_5 | 5 | 4955 | 5,000 | 0.547 | 1.045 |
| cluster_8 | T3_3000_8 | 8 | 2965 | 5,000 | 0.341 | 2.365 |
| cluster_8 | T15_3000_8 | 8 | 2965 | 5,000 | 0.508 | 1.197 |
| cluster_8 | T9_3000_8 | 8 | 2965 | 5,000 | 0.399 | 2.236 |
| cluster_8 | T21_3000_8 | 8 | 2965 | 5,000 | 0.551 | 1.157 |
| cluster_8 | T6_5000_8 | 8 | 4955 | 5,000 | 0.328 | 2.195 |
| cluster_8 | T18_5000_8 | 8 | 4955 | 5,000 | 0.508 | 1.211 |
| cluster_8 | T12_5000_8 | 8 | 4955 | 5,000 | 0.400 | 2.274 |
| cluster_8 | T24_5000_8 | 8 | 4955 | 5,000 | 0.552 | 1.159 |
| cluster_10 | T25_3000_10 | 10 | 2965 | 5,000 | 0.344 | 2.478 |
| cluster_10 | T29_3000_10 | 10 | 2965 | 5,000 | 0.511 | 1.293 |
| cluster_10 | T27_3000_10 | 10 | 2965 | 5,000 | 0.408 | 2.362 |
| cluster_10 | T31_3000_10 | 10 | 2965 | 5,000 | 0.556 | 1.281 |
| cluster_10 | T26_5000_10 | 10 | 4955 | 5,000 | 0.348 | 2.490 |
| cluster_10 | T30_5000_10 | 10 | 4955 | 5,000 | 0.502 | 1.192 |
| cluster_10 | T28_5000_10 | 10 | 4955 | 5,000 | 0.415 | 2.530 |
| cluster_10 | T32_5000_10 | 10 | 4,955 | 5,000 | 0.557 | 1.268 |

*Note:*

Sample No. : Sample number; #Cluster: Average number of clusters per sample; #Unit: Average sequenced units (e.g., cells or spots) per sample; #Gene: Average number of sequenced genes per sample; Gene.exp: Average mRNA counts per sequenced unit and gene.

**Supplementary Table 4.** **Normalized weights for self-loop and 12 nearest neighbors (k = 12) under degree normalization and DWGCN with varying distance exponent** $\boldsymbol{p}$**.**

| **Method** | **Self-loop** | **N1** | **N2** | **N3** | **N4** | **N5** | **N6** | **N7** | **N8** | **N9** | **N10** | **N11** | **N12** |
| --- | --- | --- | --- | --- | --- | --- | --- | --- | --- | --- | --- | --- | --- |
| Degree Norm | 0.077 | 0.077 | 0.077 | 0.077 | 0.074 | 0.074 | 0.072 | 0.072 | 0.072 | 0.069 | 0.067 | 0.065 | 0.064 |
| DWGCN  (p = 0) | 0.077 | 0.077 | 0.077 | 0.077 | 0.077 | 0.077 | 0.077 | 0.077 | 0.077 | 0.077 | 0.077 | 0.077 | 0.077 |
| DWGCN  (p = 0.5) | 0.121 | 0.085 | 0.085 | 0.085 | 0.085 | 0.073 | 0.073 | 0.070 | 0.070 | 0.063 | 0.063 | 0.063 | 0.063 |
| DWGCN  (p = 1) | 0.182 | 0.091 | 0.091 | 0.091 | 0.091 | 0.067 | 0.067 | 0.061 | 0.061 | 0.050 | 0.050 | 0.050 | 0.050 |
| DWGCN  (p = 2) | 0.360 | 0.090 | 0.089 | 0.089 | 0.089 | 0.048 | 0.048 | 0.040 | 0.040 | 0.027 | 0.027 | 0.027 | 0.027 |
| DWGCN  (p = 4) | 0.752 | 0.047 | 0.047 | 0.046 | 0.046 | 0.013 | 0.013 | 0.009 | 0.009 | 0.004 | 0.004 | 0.004 | 0.004 |
| DWGCN  (p = 8) | 0.984 | 0.004 | 0.004 | 0.004 | 0.004 | 0.000 | 0.000 | 0.000 | 0.000 | 0.000 | 0.000 | 0.000 | 0.000 |

***Note:***
Degree Norm represents the normalized edge weights of adjacency matrix; DWGCN

(p = 0~8) represents the distance-weighted adjacency matrix constructed by DWGCN with varying distance exponent $p$.

**Supplementary Table 5. Average values of three clustering evaluation metrics across four real datasets**

| Metrics | Dataset | SEDR | | GraphST | | SpaGIC | | SpaNCMG | |
| --- | --- | --- | --- | --- | --- | --- | --- | --- | --- |
|  |  | SEDR | DW_SEDR | GraphST | DW_GraphST | SpaGIC | DW_SpaGIC | SpaNCMG | DW_SpaNCMG |
| ARI | DLPFC | 0.505 | 0.507 | 0.484 | **0.519** | 0.412 | **0.435** | 0.334 | **0.436** |
|  | Human_Breast | 0.439 | 0.424 | 0.524 | **0.546** | 0.502 | 0.517 | 0.439 | **0.525** |
|  | Mouse_Brain | 0.396 | 0.387 | 0.401 | **0.424** | 0.388 | **0.415** | 0.342 | **0.391** |
|  | Mouse_Embryos | 0.391 | 0.394 | 0.354 | **0.364** | 0.366 | 0.362 | 0.288 | **0.418** |
| NMI | DLPFC | 0.641 | 0.638 | 0.639 | **0.658** | 0.585 | 0.591 | 0.487 | **0.598** |
|  | Human_Breast | 0.654 | 0.647 | 0.673 | **0.681** | 0.679 | 0.678 | 0.518 | **0.67** |
|  | Mouse_Brain | 0.702 | 0.7 | 0.703 | **0.708** | **0.723** | 0.72 | 0.672 | **0.727** |
|  | Mouse_Embryos | 0.583 | 0.584 | 0.542 | 0.547 | 0.549 | **0.562** | 0.468 | **0.588** |
| Homogeneity | DLPFC | 0.669 | 0.663 | 0.632 | **0.672** | 0.626 | 0.63 | 0.509 | **0.634** |
|  | Human_Breast | **0.666** | 0.658 | 0.671 | **0.685** | 0.697 | 0.696 | 0.524 | **0.687** |
|  | Mouse_Brain | 0.725 | 0.721 | 0.688 | **0.708** | **0.751** | 0.744 | 0.692 | **0.753** |
|  | Mouse_Embryos | 0.583 | 0.579 | 0.515 | **0.523** | 0.551 | **0.56** | 0.464 | **0.588** |

Note:
This table reports the average values of three clustering evaluation metrics (ARI, NMI, and Homogeneity) across four real datasets for four graph-based spatial transcriptomics methods (SEDR, GraphST, SpaGIC, and SpaNCMG). Each method was evaluated in its original form and its DWGCN-enhanced version.
The higher metric value within each method pair is underlined. If the difference between the two variants is statistically significant (paired rank test, p < 0.05), the better result is also **bolded**.

**Supplementary Table 6. Paired difference and effect size analysis of real datasets**

| Metrics | Dataset | $\boldsymbol{\Delta}\boldsymbol{value}$ | | | | Cliff Delta ($\delta$) | | | |
| --- | --- | --- | --- | --- | --- | --- | --- | --- | --- |
|  |  | SEDR | GraphST | SpaGIC | SpaNCMG | SEDR | GraphST | SpaGIC | SpaNCMG |
| ARI | DLPFC | 0.003 | **0.035** | **0.023** | **0.102** | 0.03 | **0.58** | 0.31 | **0.87** |
|  | Human_Breast | -0.015 | **0.022** | 0.015 | **0.085** | -0.31 | **0.68** | 0.35 | **1.00** |
|  | Mouse_Brain | -0.009 | **0.022** | **0.028** | **0.049** | -0.17 | **0.91** | **0.88** | **0.95** |
|  | Mouse_Embryos | 0.003 | **0.010** | -0.003 | **0.129** | 0.17 | **0.52** | -0.02 | **1.00** |
| NMI | DLPFC | -0.003 | **0.018** | 0.007 | **0.111** | **-0.07** | **0.58** | **0.08** | **0.99** |
|  | Human_Breast | -0.006 | **0.007** | -0.001 | **0.152** | **-0.52** | **0.82** | **-0.19** | **1.00** |
|  | Mouse_Brain | -0.003 | **0.005** | **-0.003** | **0.055** | **-0.39** | **0.94** | **-0.77** | **1.00** |
|  | Mouse_Embryos | 0.002 | 0.005 | **0.012** | **0.120** | **0.11** | **0.31** | **0.47** | **1.00** |
| Homogeneity | DLPFC | -0.006 | **0.040** | 0.004 | **0.126** | **-0.14** | **0.79** | **0.01** | **1.00** |
|  | Human_Breast | **-0.008** | **0.015** | 0.000 | **0.162** | **-0.66** | **0.94** | **-0.09** | **1.00** |
|  | Mouse_Brain | -0.003 | **0.019** | **-0.007** | **0.062** | **-0.46** | **1.00** | **-0.97** | **1.00** |
|  | Mouse_Embryos | -0.004 | **0.007** | **0.010** | **0.125** | **-0.29** | **0.39** | **0.42** | **1.00** |

Note:

This table summarizes the average value of paired differences (
 $\boldsymbol{\Delta}\boldsymbol{value}$) between the *DWGCN-enhanced* and original methods for three metrics (ARI, NMI, and Homogeneity). A positive $\boldsymbol{\Delta}\boldsymbol{value}$ indicates improved performance of the *DWGCN-enhanced* version. Statistically significant improvements (*FDR* *< 0.05*, paired rank testing) are highlighted in **bolded**. The *Cliff’s Delta* values ($\delta$) quantify effect size; large effects (|δ| ≥ 0.47) are highlighted in bolded, following standard interpretation thresholds (Negligible: |δ| < 0.15; small: 0.15 ≤ |δ| < 0.33; medium: 0.33 ≤ |δ| < 0.47; large: |δ| ≥ 0.47).

**Supplementary Table 7. Summary of DWGCN sample-level improvement over four benchmark methods on real datasets.**

| Metric | Class | SEDR | GraphST | SpaGIC | SpaNCMG | ALL |
| --- | --- | --- | --- | --- | --- | --- |
| ARI | improve | 41.18% (7/17) | 88.24% (15/17) | 76.47% (13/17) | 94.12% (16/17) | 75% (51/68) |
| ARI | significant.improve | 5.88% (1/17) | 64.71% (11/17) | 29.41% (5/17) | 82.35% (14/17) | 45.59% (31/68) |
| ARI | decrease | 58.82% (10/17) | 11.76% (2/17) | 23.53% (4/17) | 5.88% (1/17) | 25% (17/68) |
| ARI | significant.decrease | 0% (0/17) | 11.76% (2/17) | 5.88% (1/17) | 0% (0/17) | 4.41% (3/68) |
| NMI | improve | 35.29% (6/17) | 88.24% (15/17) | 47.06% (8/17) | 100% (17/17) | 67.65% (46/68) |
| NMI | significant.improve | 0% (0/17) | 58.82% (10/17) | 23.53% (4/17) | 100% (17/17) | 45.59% (31/68) |
| NMI | decrease | 64.71% (11/17) | 11.76% (2/17) | 52.94% (9/17) | 0% (0/17) | 32.35% (22/68) |
| NMI | significant.decrease | 5.88% (1/17) | 11.76% (2/17) | 17.65% (3/17) | 0% (0/17) | 8.82% (6/68) |
| Homogeneity | improve | 29.41% (5/17) | 100% (17/17) | 47.06% (8/17) | 100% (17/17) | 69.12% (47/68) |
| Homogeneity | significant.improve | 5.88% (1/17) | 70.59% (12/17) | 23.53% (4/17) | 100% (17/17) | 50% (34/68) |
| Homogeneity | decrease | 70.59% (12/17) | 0% (0/17) | 52.94% (9/17) | 0% (0/17) | 30.88% (21/68) |
| Homogeneity | significant.decrease | 5.88% (1/17) | 0% (0/17) | 35.29% (6/17) | 0% (0/17) | 10.29% (7/68) |

Note:

This table summarizes the percentage of datasets in which DWGCN outperforms or underperforms four benchmark methods across three clustering evaluation metrics.
“improve” indicates cases where DWGCN achieves a higher score than the compared method; “significant.improve” denotes statistically significant improvement (*FDR* < 0.05, Wilcoxon signed-rank test); “decrease” and “significant.decrease” represent lower and significantly lower performance, respectively.
The “ALL” column reports the aggregated proportions across all 68 pairwise comparisons (17 samples × 4 baseline methods).

**Supplementary Table 8. Average values of three clustering evaluation metrics across four simulated datasets**

| Metrics | Dataset | SEDR | | GraphST | | SpaGIC | | SpaNCMG | |
| --- | --- | --- | --- | --- | --- | --- | --- | --- | --- |
|  |  | SEDR | DW_SEDR | GraphST | DW_GraphST | SpaGIC | DW_SpaGIC | SpaNCMG | DW_SpaNCMG |
| ARI | cluster_3 | 0.954 | **0.965** | **0.757** | 0.672 | 0.822 | **0.857** | 0.549 | 0.561 |
|  | cluster_5 | 0.731 | **0.862** | 0.424 | 0.437 | 0.445 | 0.447 | 0.241 | **0.362** |
|  | cluster_8 | 0.39 | **0.497** | 0.136 | **0.212** | 0.253 | **0.269** | 0.122 | **0.189** |
|  | cluster_10 | 0.33 | **0.436** | 0.076 | **0.133** | 0.223 | **0.247** | 0.107 | **0.168** |
| NMI | cluster_3 | 0.901 | **0.924** | **0.695** | 0.626 | 0.762 | **0.836** | **0.641** | 0.619 |
|  | cluster_5 | 0.756 | **0.825** | 0.540 | 0.551 | 0.573 | **0.622** | 0.487 | **0.536** |
|  | cluster_8 | 0.621 | **0.679** | 0.434 | **0.485** | 0.484 | **0.540** | 0.443 | **0.462** |
|  | cluster_10 | 0.622 | **0.667** | 0.432 | **0.477** | 0.491 | **0.548** | 0.448 | **0.465** |
| Homogeneity | cluster_3 | 0.894 | **0.917** | **0.691** | 0.614 | 0.767 | **0.852** | **0.676** | 0.653 |
|  | cluster_5 | 0.800 | **0.837** | 0.586 | **0.597** | 0.646 | **0.710** | 0.544 | **0.608** |
|  | cluster_8 | 0.719 | **0.764** | 0.490 | **0.552** | 0.571 | **0.641** | 0.518 | **0.545** |
|  | cluster_10 | 0.713 | **0.743** | 0.471 | **0.535** | 0.570 | **0.637** | 0.510 | **0.533** |

Note:
This table reports the average values of three clustering evaluation metrics (ARI, NMI, and Homogeneity) across four simulated datasets for four graph-based spatial transcriptomics methods (SEDR, GraphST, SpaGIC, and SpaNCMG). Each method was evaluated in its original form and its DWGCN-enhanced version.
The higher metric value within each method pair is underlined. If the difference between the two variants is statistically significant (*FDR* < 0.05), the better result is also **bolded**.

**Supplementary Table 9. Paired difference and effect size analysis of simulated datasets**

| Metrics | Dataset | $\Delta Value$ | | | | Cliff Delta ($\delta$) | | | |
| --- | --- | --- | --- | --- | --- | --- | --- | --- | --- |
|  |  | SEDR | GraphST | SpaGIC | SpaNCMG | SEDR | GraphST | SpaGIC | SpaNCMG |
| ARI | cluster_3 | **0.011** | **-0.085** | **0.035** | 0.012 | **0.99** | -0.27 | **0.57** | -0.08 |
|  | cluster_5 | **0.131** | 0.013 | 0.002 | **0.121** | **0.68** | 0.07 | 0.10 | **0.85** |
|  | cluster_8 | **0.107** | **0.076** | **0.016** | **0.067** | **0.71** | **0.70** | 0.29 | **0.87** |
|  | cluster_10 | **0.106** | **0.057** | **0.025** | **0.062** | **0.81** | **0.79** | **0.44** | **0.94** |
| NMI | cluster_3 | **0.023** | **-0.069** | **0.075** | **-0.022** | **1.00** | **-0.29** | **0.81** | **-0.46** |
|  | cluster_5 | **0.069** | 0.011 | **0.049** | **0.048** | **0.72** | **0.16** | **0.71** | **0.76** |
|  | cluster_8 | **0.058** | **0.051** | **0.056** | **0.019** | **0.88** | **0.85** | **0.92** | **0.60** |
|  | cluster_10 | **0.045** | **0.045** | **0.057** | **0.017** | **0.87** | **0.84** | **1.00** | **0.72** |
| Homogeneity | cluster_3 | **0.023** | **-0.077** | **0.085** | **-0.023** | **1.00** | **-0.28** | **0.83** | **-0.56** |
|  | cluster_5 | **0.037** | **0.011** | **0.064** | **0.064** | **0.68** | **0.46** | **0.88** | **0.83** |
|  | cluster_8 | **0.045** | **0.062** | **0.071** | **0.027** | **0.89** | **0.85** | **0.95** | **0.61** |
|  | cluster_10 | **0.031** | **0.064** | **0.067** | **0.023** | **0.82** | **0.86** | **0.99** | **0.71** |

Note:

This table summarizes the average value of paired differences (
 $\boldsymbol{\Delta}\boldsymbol{value}$) between the *DWGCN-enhanced* and original methods for three metrics (ARI, NMI, and Homogeneity). A positive $\boldsymbol{\Delta}\boldsymbol{value}$ indicates improved performance of the *DWGCN-enhanced* version. Statistically significant improvements (*FDR* *< 0.05*, paired rank testing) are highlighted in **bolded**. The *Cliff’s Delta* values ($\delta$) quantify effect size; large effects (|δ| ≥ 0.47) are highlighted in bolded, following standard interpretation thresholds (Negligible: |δ| < 0.15; small: 0.15 ≤ |δ| < 0.33; medium: 0.33 ≤ |δ| < 0.47; large: |δ| ≥ 0.47).

**Supplementary Table 10. Summary of DWGCN sample-level improvement over four benchmark methods on simulated datasets.**

| Metric | Class | SEDR | GraphST | SpaGIC | SpaNCMG | ALL |
| --- | --- | --- | --- | --- | --- | --- |
| ARI | improve | 93.75% (30/32) | 78.12% (25/32) | 75% (24/32) | 90.62% (29/32) | 84.38% (108/128) |
| ARI | significant.improve | 78.12% (25/32) | 43.75% (14/32) | 34.38% (11/32) | 75% (24/32) | 57.81% (74/128) |
| ARI | decrease | 6.25% (2/32) | 21.88% (7/32) | 25% (8/32) | 9.38% (3/32) | 15.62% (20/128) |
| ARI | significant.decrease | 0% (0/32) | 9.38% (3/32) | 9.38% (3/32) | 0% (0/32) | 4.69% (6/128) |
| NMI | improve | 100% (32/32) | 81.25% (26/32) | 100% (32/32) | 75% (24/32) | 89.06% (114/128) |
| NMI | significant.improve | 96.88% (31/32) | 43.75% (14/32) | 84.38% (27/32) | 46.88% (15/32) | 67.97% (87/128) |
| NMI | decrease | 0% (0/32) | 18.75% (6/32) | 0% (0/32) | 25% (8/32) | 10.94% (14/128) |
| NMI | significant.decrease | 0% (0/32) | 9.38% (3/32) | 0% (0/32) | 6.25% (2/32) | 3.91% (5/128) |
| Homogeneity | improve | 100% (32/32) | 78.12% (25/32) | 100% (32/32) | 75% (24/32) | 88.28% (113/128) |
| Homogeneity | significant.improve | 93.75% (30/32) | 50% (16/32) | 96.88% (31/32) | 50% (16/32) | 72.66% (93/128) |
| Homogeneity | decrease | 0% (0/32) | 21.88% (7/32) | 0% (0/32) | 25% (8/32) | 11.72% (15/128) |
| Homogeneity | significant.decrease | 0% (0/32) | 9.38% (3/32) | 0% (0/32) | 9.38% (3/32) | 4.69% (6/128) |

Note:

This table summarizes the percentage of datasets in which DWGCN outperforms or underperforms four benchmark methods across three clustering evaluation metrics.
“improve” indicates cases where DWGCN achieves a higher score than the compared method; “significant.improve” denotes statistically significant improvement (*FDR* < 0.05, Wilcoxon signed-rank test); “decrease” and “significant.decrease” represent lower and significantly lower performance, respectively.
The “ALL” column reports the aggregated proportions across all 128 pairwise comparisons (32 samples × 4 baseline methods).

# Supplementary Figures


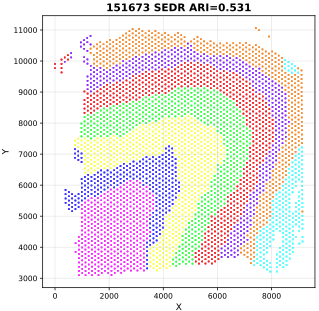

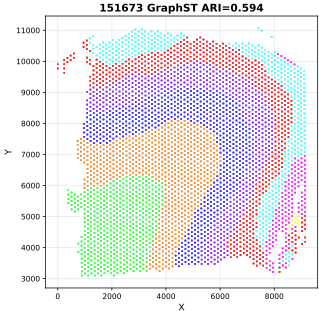

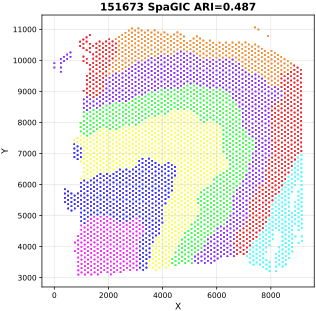

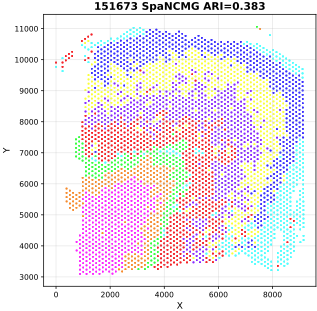


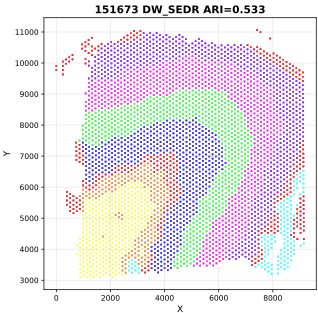

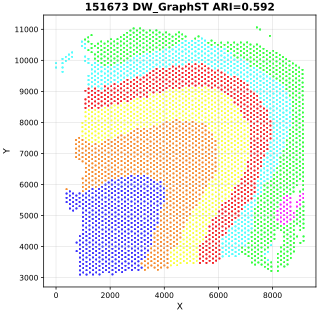

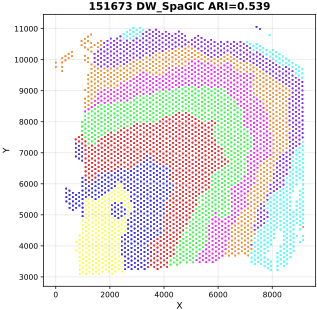

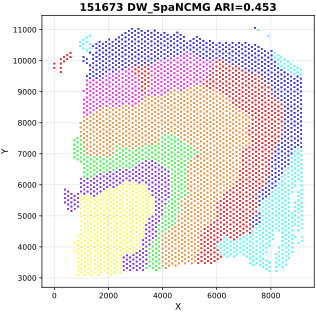


**Supplementary Figure 1.** **Spatial domain identification of 151673 from the DLPFC dataset.** Comparisons of the annotations of the original and DWGCN enhanced methods using SEDR, GraphST, SpaGIC and SpaNCMG.


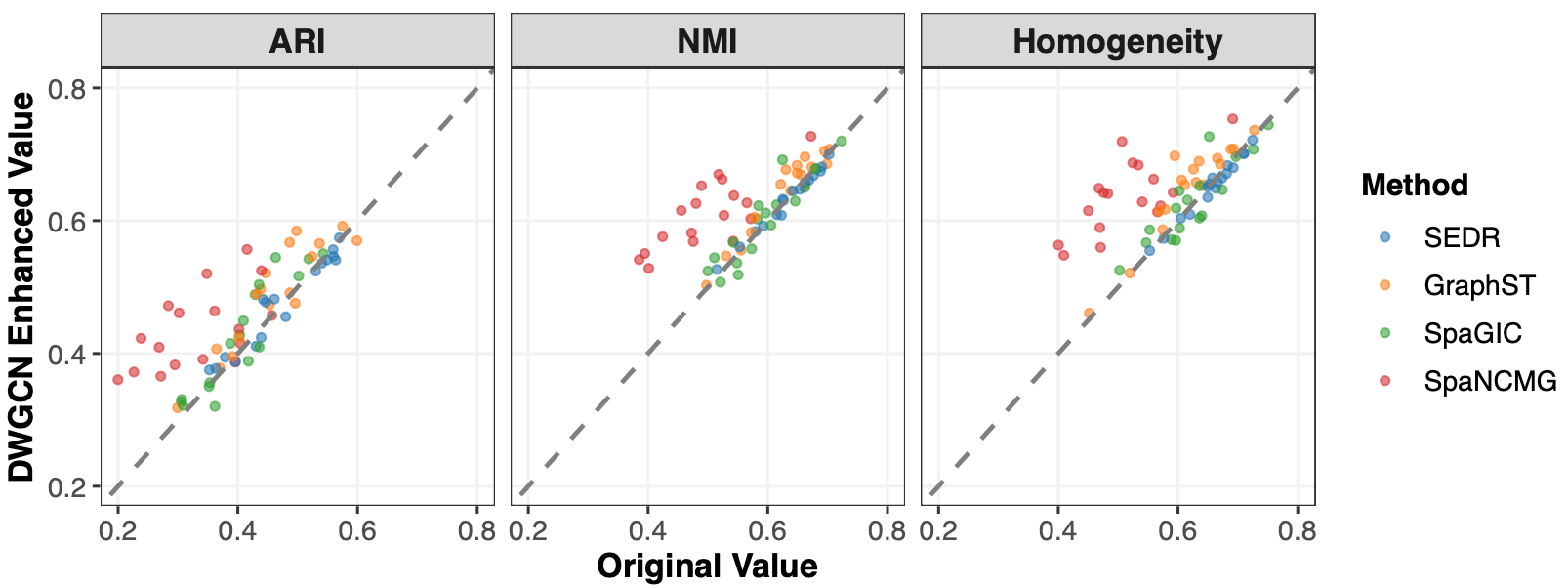


**Supplementary Figure 2. Scatter plots of ARI, NMI, and Homogeneity for real datasets.**

Each point corresponds to one sample; the x-axis reports the original method’s mean score, while the y-axis reports the DWGCN-enhanced mean score. Colors represent different baseline methods. Most points lie above the diagonal line, indicating consistent performance gains after DWGCN enhancement across all three-evaluation metrics.


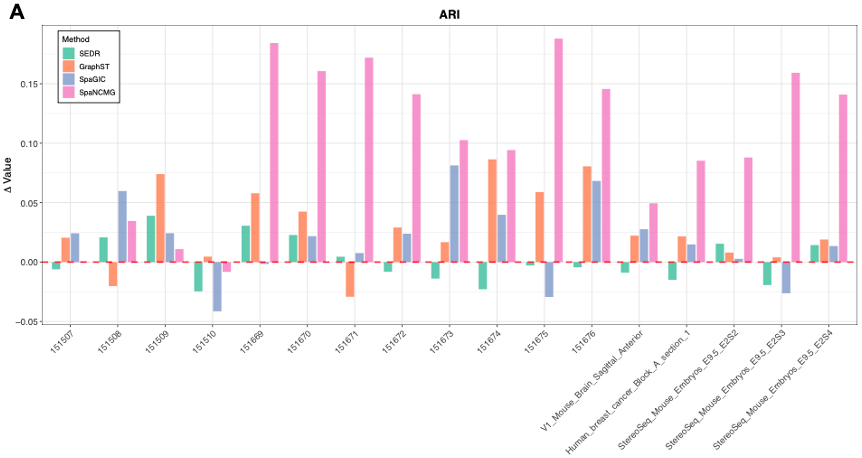


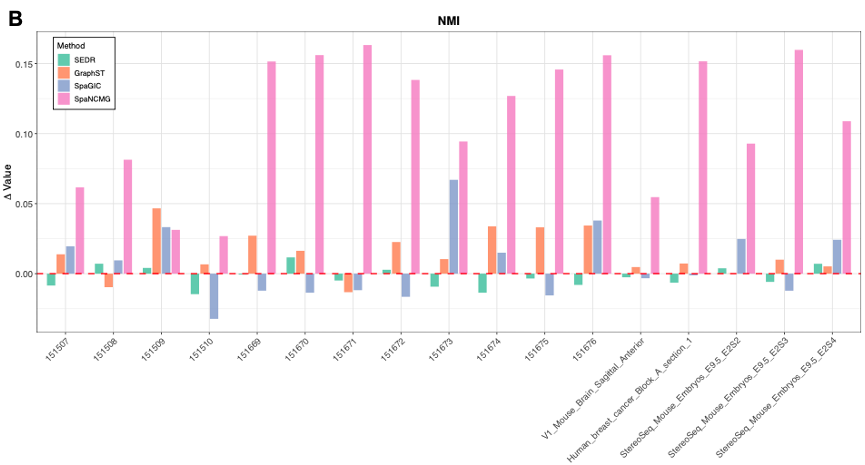


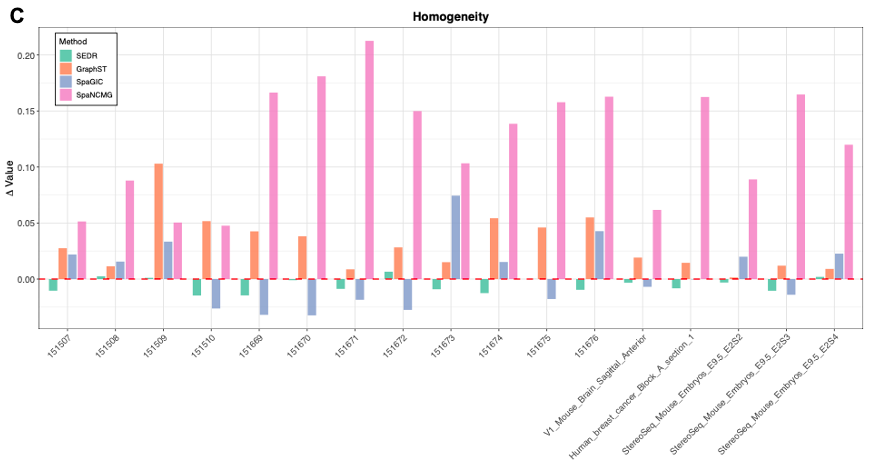


**Supplementary Figure 3. Performance improvement on real datasets following DWGCN enhancement.** Bar plots showing sample-wise changes in ARI, NMI, and Homogeneity ($\boldsymbol{\Delta}\boldsymbol{value}$) for real datasets. Positive $\boldsymbol{\Delta}\boldsymbol{value}$ dominate across samples, confirming that distance-aware edge weighting systematically improves spatial domain identification on real tissue data.


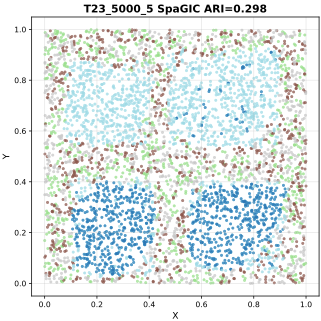

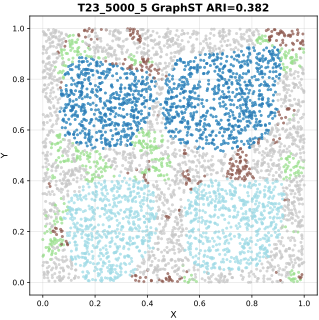

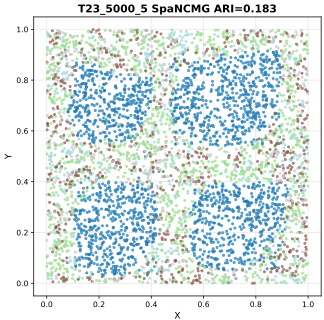

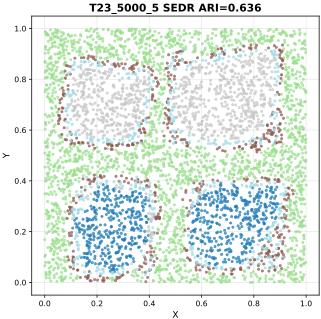


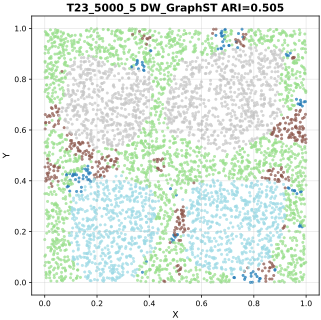

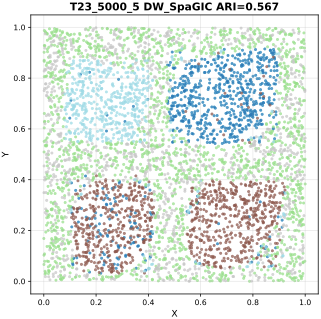

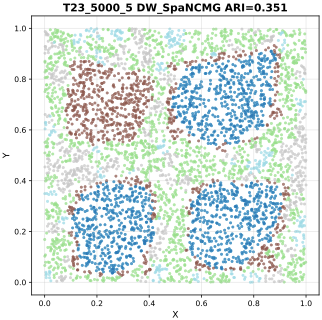

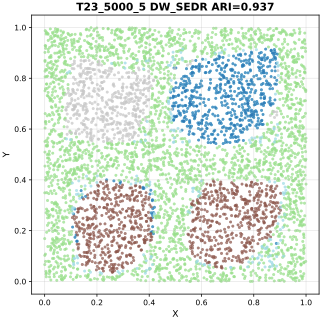


**Supplementary Figure 4. Spatial domain identification on sample T23_5000_5 of Cluster 5 dataset using original methods and DWGCN enhanced methods.**


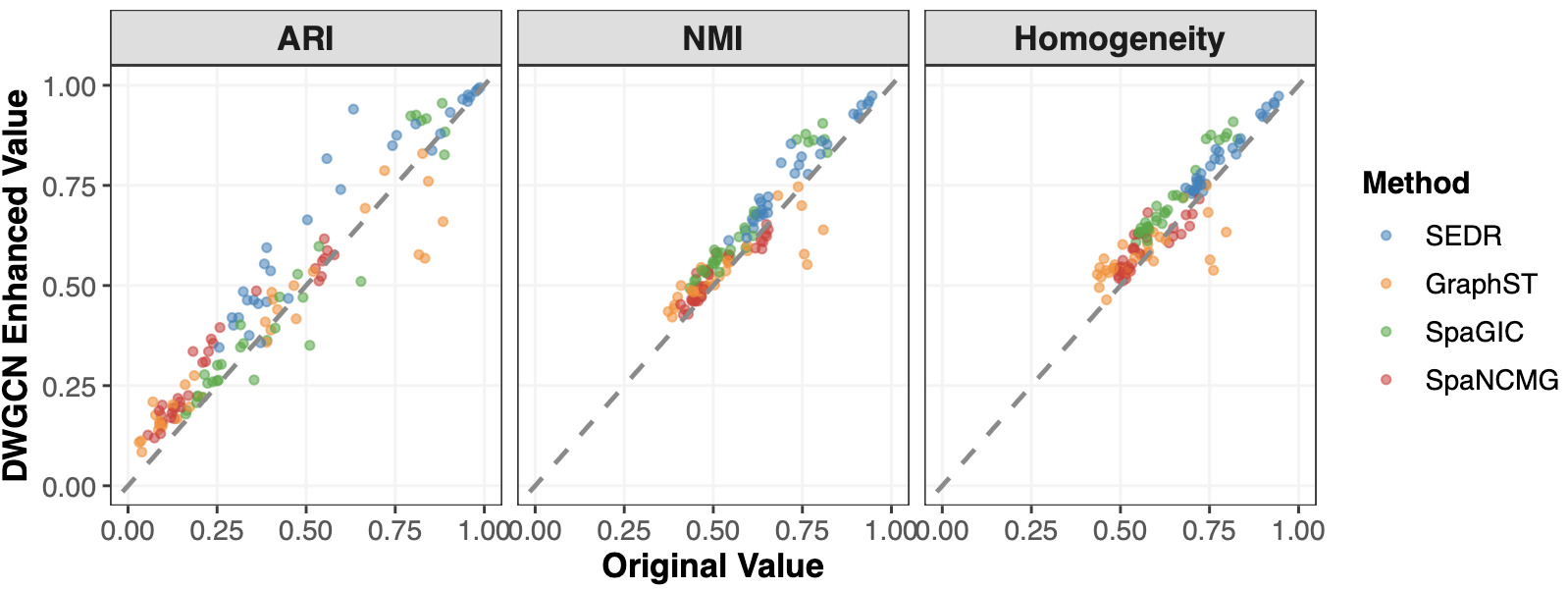


**Supplementary Figure 5. Scatter plots of ARI, NMI, and Homogeneity for simulated datasets.** Each point corresponds to one sample; The x-axis denotes the original method’s mean performance, and the y-axis denotes the DWGCN-enhanced performance. Colors represent different baseline methods. Most points lie above the diagonal line, indicating consistent performance gains after DWGCN enhancement across all three-evaluation metrics.


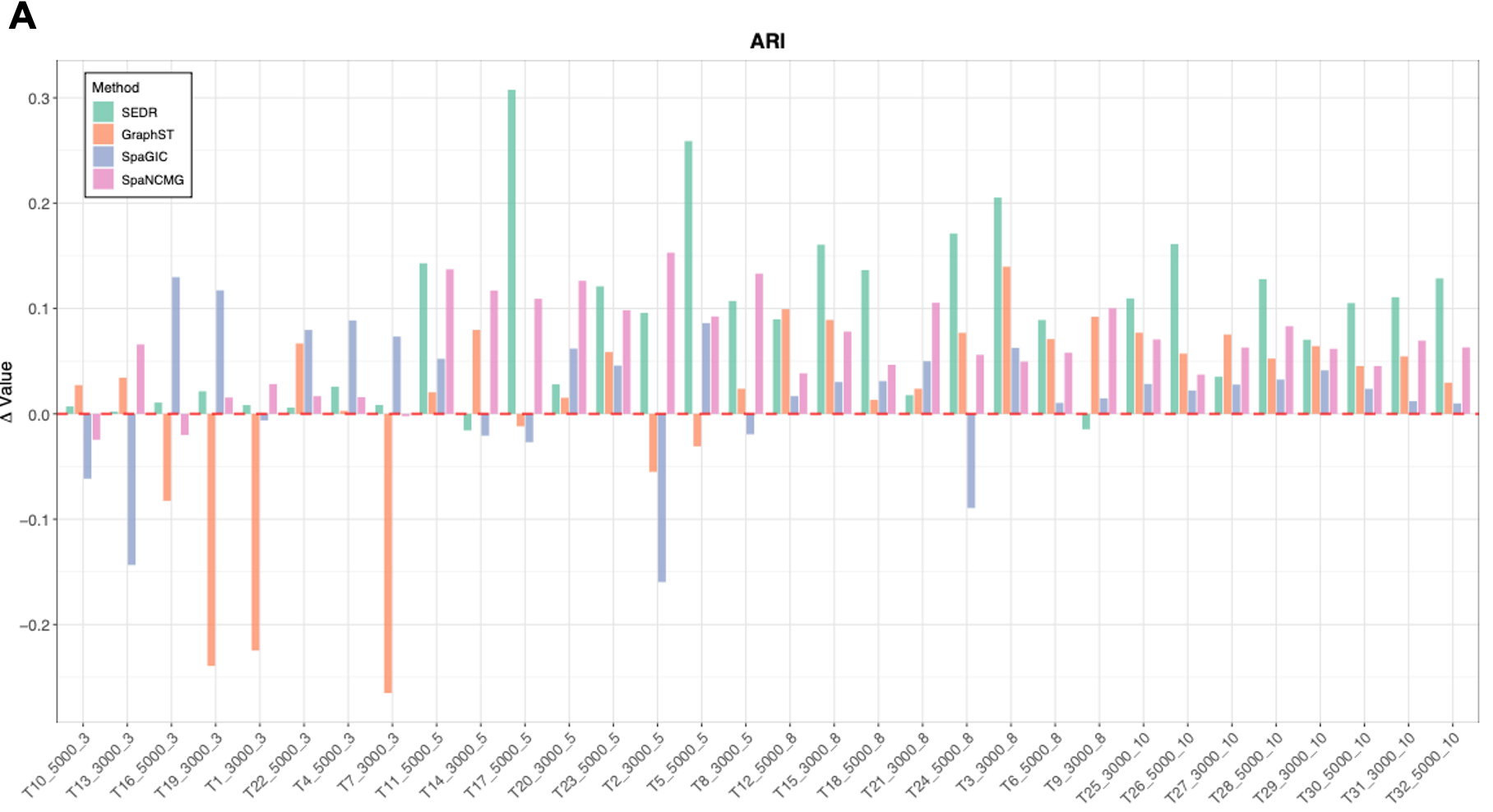


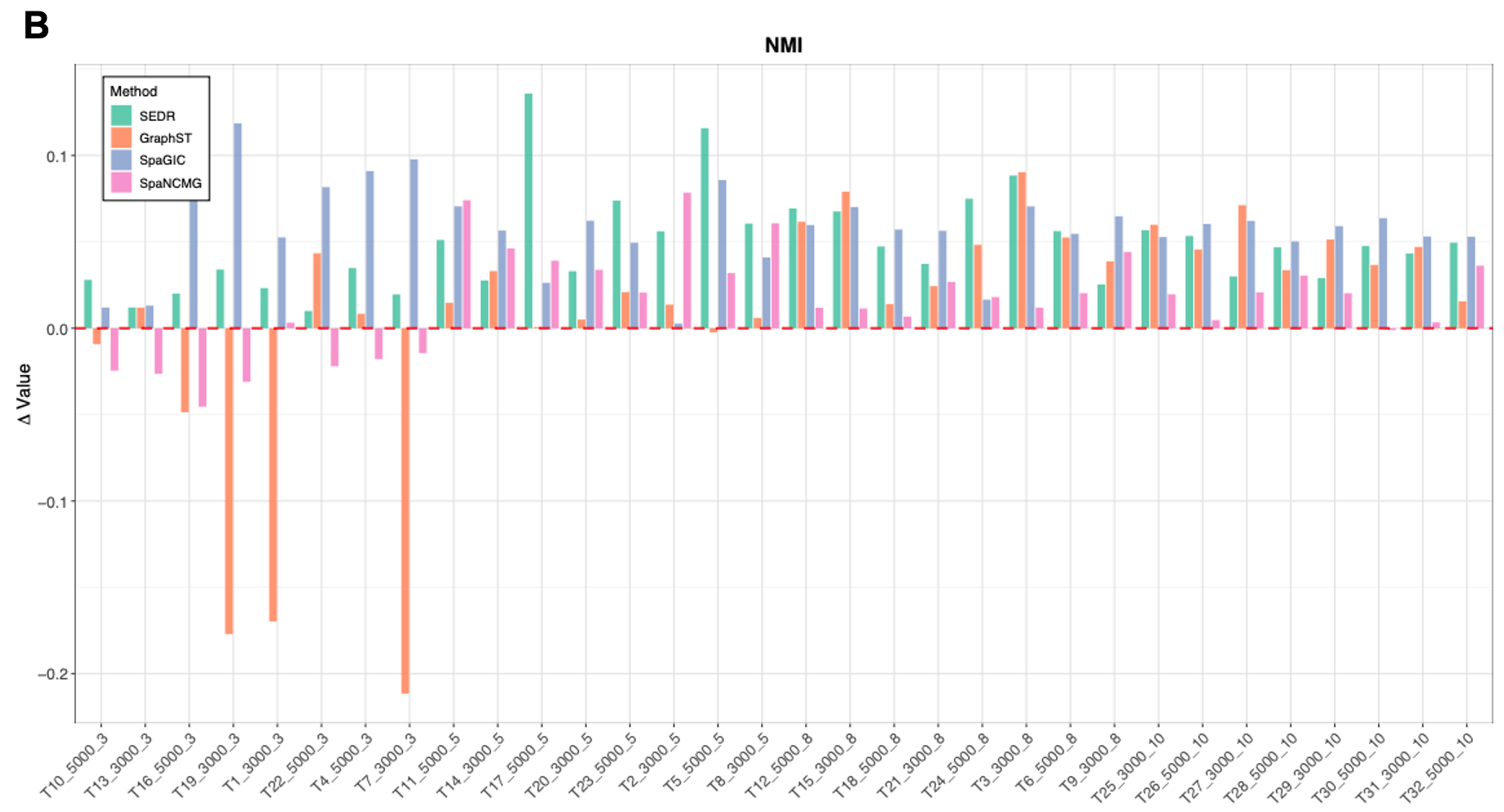


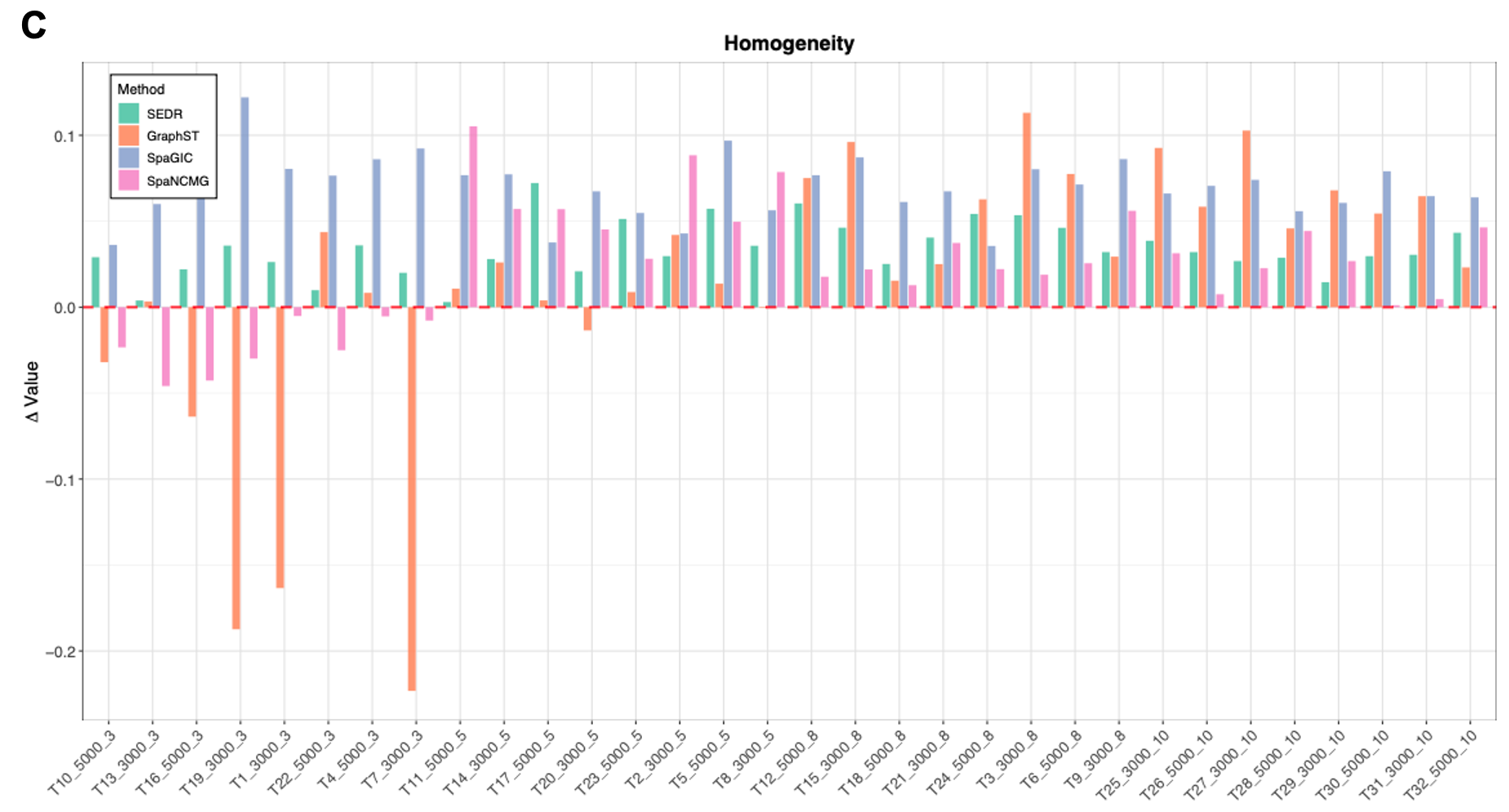


**Supplementary Figure 6. Performance improvement on simulated datasets following DWGCN enhan**cement. Bar plots of $\boldsymbol{\Delta}\boldsymbol{value}$ of ARI, NMI, and Homogeneity across all simulated datasets. Positive $\boldsymbol{\Delta}\boldsymbol{value}$ dominate across samples, confirming that distance-aware edge weighting systematically improves spatial domain identification on real tissue data. Across baseline methods, DWGCN provides stable and predominantly positive gains, validating its effectiveness under simulated noise patterns and spatial configurations.
